# The transcriptional landscape of *Chlamydia pneumoniae*

**DOI:** 10.1186/gb-2011-12-10-r98

**Published:** 2011-10-11

**Authors:** Marco Albrecht, Cynthia M Sharma, Marcus T Dittrich, Tobias Müller, Richard Reinhardt, Jörg Vogel, Thomas Rudel

**Affiliations:** 1 Department of Microbiology, Biocenter, University of Würzburg, Am Hubland, Würzburg, 97074, Germany; 2 Research Center for Infectious Diseases, University of Würzburg, Joseph Schneider Str. 2, Würzburg, 97080, Germany; 3 Department of Bioinformatics, Biocenter, University of Würzburg, 97074, Würzburg, Germany; 4 Max Planck Genome Centre Cologne, Max Planck Institute for Plant Breeding Research, Carl-von-Linné-Weg 10, Cologne, 50829, Germany; 5 Institute for Molecular Infection Biology, University of Würzburg, Würzburg, 97080, Germany

## Abstract

**Background:**

Gene function analysis of the obligate intracellular bacterium *Chlamydia pneumoniae *is hampered by the facts that this organism is inaccessible to genetic manipulations and not cultivable outside the host. The genomes of several strains have been sequenced; however, very little information is available on the gene structure and transcriptome of *C. pneumoniae*.

**Results:**

Using a differential RNA-sequencing approach with specific enrichment of primary transcripts, we defined the transcriptome of purified elementary bodies and reticulate bodies of *C. pneumoniae *strain CWL-029; 565 transcriptional start sites of annotated genes and novel transcripts were mapped. Analysis of adjacent genes for co-transcription revealed 246 polycistronic transcripts. In total, a distinct transcription start site or an affiliation to an operon could be assigned to 862 out of 1,074 annotated protein coding genes. Semi-quantitative analysis of mapped cDNA reads revealed significant differences for 288 genes in the RNA levels of genes isolated from elementary bodies and reticulate bodies. We have identified and in part confirmed 75 novel putative non-coding RNAs. The detailed map of transcription start sites at single nucleotide resolution allowed for the first time a comprehensive and saturating analysis of promoter consensus sequences in *Chlamydia*.

**Conclusions:**

The precise transcriptional landscape as a complement to the genome sequence will provide new insights into the organization, control and function of genes. Novel non-coding RNAs and identified common promoter motifs will help to understand gene regulation of this important human pathogen.

## Background

The human pathogen *Chlamydia pneumoniae *(*Cpn*; also referred to as *Chlamydophila pneumoniae *[1]) is a major cause of pneumonia and chronic infection has also been associated with atherosclerosis [2] and Alzheimer's disease [3]. *Cpn *can cause a spectrum of infections that usually take a mild or sub-clinical course. It causes acute respiratory disease [4] and accounts for 6 to 20% of community-acquired pneumonia cases in adults [5]. Almost all humans can expect to be infected with *Cpn *at least once during their lifetime and infections can become chronic. Re-infections during the lifetime are common, leading to a seroprevalence of 80% in adults [6]. *Cpn *is an obligate intracellular Gram-negative bacteria with a unique biphasic developmental cycle [7]. The infection starts with the endocytic uptake of the metabolically inactive elementary bodies (EBs) by the eukaryotic cell [8]. EBs differentiate to metabolically active reticulate bodies (RBs), which replicate in a vacuole inside the host cell. RBs re-differentiate to EBs, which are then released from the cells to initiate a new cycle of infection. Currently, no vaccine is available to prevent *Cpn *infection; however, acute infections can be treated with antibiotics like macrolines and doxycycline. Atypical persistent inclusions are resistant to antibiotic treatment and seropositivity for *Cpn *correlates with increased lung cancer risk [9].

Since genetic tools to manipulate the genome and methods to culture the bacteria outside the host cell are lacking, genome sequence analysis has been the main approach to gain insight into the biology of all Chlamydiales. The genome sequence of *Cpn *has been available since 1999 [10] and most information on the gene organization of this organism is based on comparative genome analysis. *Cpn *strain CWL-029 harbors a circular chromosome of 1,230,230 nucleotides (GC content 40%, coding capacity 88%) that is predicted to carry 1,122 genes, including 1,052 protein coding genes [10]. The biphasic life cycle is unique to *Chlamydia *and is probably controlled by differential regulation of multiple genes since gene expression patterns vary enormously between the life cycle stages [11]. However, very little information is available about gene regulation in *Cpn *and most of the data on promoter structures and functions has been obtained in heterologous systems. Alternative RNA polymerases might be used to control gene expression. Besides the major sigma factor σ^66 ^(homologous to the *Escherichia coli *housekeeping σ^70^), two alternative sigma factors have been identified in the genome but their functions are largely unknown. Chlamydial σ^28 ^is a homologue of *E. coli *σ^28 ^and belongs to the group of σ^70 ^factors. The third chlamydial sigma factor, σ^54^, has been suggested to be developmentally regulated by the sensory kinase AtoS and the response regulator AtoC [12].

The function of the three σ factors is largely unknown. Studies on temporal expression patterns of the *Chlamydia trachomatis *(*Ctr*) σ factor genes are controversial. Douglas and Hatch [13] did not detect differences in the σ factor expression patterns throughout the chlamydial life cycle whereas Matthews *et al*. [14] reported an early stage expression of *rpoD *and a mid- and late-stage expression of of *rpsD *and *rpoN*. Detailed studies on *Cpn *σ factor genes are not available so far. The RNA polymerase core enzyme genes and the major σ factor gene *rpoD *are expressed at relatively constant levels during the whole developmental cycle [13]. This is consistent with the expected function of regulating housekeeping genes. Promoter motifs have been predicted computationally based on their homology to the σ^70 ^family promoters. Several σ^70 ^target genes such as *ompA *and *omcB*, could be verified experimentally [15]. The role of the two alternative σ factors is still unknown but some of the late genes expressed at the stage of RB-to-EB conversion seem to be directly regulated by σ^28 ^[16-18].

Recently, small non-coding RNAs (sRNAs) were identified as a group of regulatory molecules in all species in which they have been searched for. They are acting at all layers of gene regulation, that is, transcription, mRNA stability and protein activity (reviewed in [19]). Additionally, proteins have been identified that mediate the interaction of sRNAs with their targets. In bacteria, most sRNAs coordinate adaptation processes in response to environmental signals [20]. So far, no sRNA as well as no homologue of the conserved RNA chaperone Hfq have been reported for *Cpn *but recent studies identified numerous sRNAs in *Ctr *[21-23]. The strong inter-species homology of *Chlamydia *suggests that *Cpn *also contains a set of sRNAs. We recently used a differential RNA sequencing approach (dRNA-seq [24]) to map the primary transcriptome of *Ctr *and thereby identified hundreds of transcription start sites (TSSs) and several sRNAs [21]. Despite the high degree of homology at the genome level, the comparative analysis of *Cpn *and *Ctr *revealed major differences in gene organization and differential expression between EBs and RBs.

Here we used dRNA-seq to map the transcriptome of purified EBs and RBs. Applying an enzymatic enrichment for RNA molecules with native 5' triphosphate [24], we could map TSSs of annotated genes and novel transcripts comprising candidate non-coding RNAs that are located in intergenic regions and antisense to annotated ORFs. Furthermore, polycistronic transcripts have been identified and promoter consensus sequences based on defined TSSs have been predicted. Our data provide novel insights into the gene structures of *Cpn *and a comprehensive landscape of EB and RB gene activity. The annotated primary transcriptome of *Cpn*, including a comprehensive list of candidate sRNAs, will help to understand gene regulation of this important genetically intractable pathogen.

## Results and discussion

### dRNA-seq of *C. pneumoniae*

In order to determine the transcriptome of *Cpn *at different developmental stages, EBs and RBs were purified from discontinuous sucrose gradients and the purity of EB and RB fractions was validated by electron microscopy (Figure S1 in Additional file 1). RNA was isolated from purified EBs and RBs for subsequent pyrosequencing of all RNAs and RNAs enriched for TSSs (see Materials and methods for details). RNA integrity was assessed by capillary electrophoresis. Absence of eukaryotic 18S and 23S ribosomal RNA in the purified EB and RB RNA served as a control for RNA purity (Figure S2A, B in Additional file 1). Northern blot analysis of RNA fractions showed no significant RNA degradation and enrichment of chlamydial RNA in the EB and RB RNA samples (Figure S2C in Additional file 1). In total 1,437,231 sequence reads were obtained from four cDNA libraries comprising more than 97 million nucleotides. Of these, 1,221,744 sequence reads (85%) of at least 18 nucleotides in length were blasted against the *Cpn *genome to yield 854,242 sequence reads (70%) that mapped to the genome (for details see Table S1 in Additional file 1). Concordant with the literature, a plasmid could not be detected in this strain. The remaining sequences were of human origin or could not be mapped to known sequences due to sequencing errors.

For 982 of the 1,122 (87.5%) genes from the genome annotation [10] at least 10 sequence reads were obtained. The most abundant protein coding genes were *omcB*, *ompA*, *hctB *and *omcA *with more than 2,000 cDNA reads per locus. Of the genes that were covered by less than ten sequence reads per gene, 69% were genes of unknown function. These genes were either expressed at low levels under the conditions applied or seem to be wrongly annotated. Sequence reads located in intergenic regions or antisense to annotated genes, including candidates for non-protein-coding RNAs, account for 8.5% of all sequence reads obtained.

**Figure 1:**
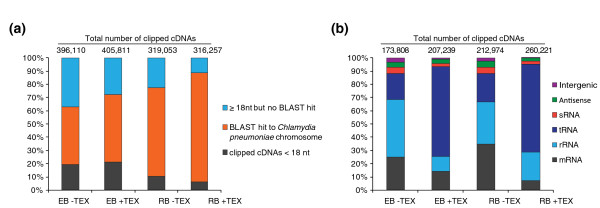
**Classification of sequencing reads**. Sequencing reads of at least 18 nucleotides in length were blasted against the *C. pneumonia *CWL-029 genome. **(a) **The fraction of sequences that could be mapped to the *Chlamydia *genome is given for each library. Sequence reads shorter than 17 nucleotides were not considered for BLAST search. **(b) **The distribution of sequence reads among the different classes of RNA is shown for each library. Nt, nucleotides; TEX, terminator exonuclease.

The fraction of RNA molecules shorter than 18 nucleotides was larger in the two EB libraries compared to the RB libraries (Figure 1a). Also, the fraction of cDNA reads that could not be mapped to the *Cpn *genome was significantly larger in the EB libraries. These sequences were derived from contaminating host cell RNA that was not depleted during *Chlamydia *isolation and purification. The fraction of reads that could be mapped to the genome was subdivided into the different classes of RNAs shown in Figure 1b. The fraction of mRNA reads was considerably decreased in the terminator exonuclease (TEX)-treated libraries due to the degradation of mRNA fragments lacking the tri-phosphate (5'PPP) RNA ends by TEX. Likewise, the fraction of rRNA was decreased, and that of tRNA increased upon nuclease treatment (Figure 1b).

The average sequence length of all cDNAs after 5'-end linker and polyA clipping was 68.14 nucleotides with read lengths up to 400 nucleotides (Figure S3 in Additional file 1). Peaks in the length distribution originated from abundant RNAs like tRNAs (70- to 90-nucleotide peaks) and 5S ribosomal RNA (123-nucleotide peak). The peak at 165 nucleotides was only present in the EB enriched library and derived from contaminating human U1 small nucleolar RNA.

### Annotation of transcriptional start sites

The primary annotation of the *Cpn *CWL-029 genome contains 1,122 genes, comprising 1,074 protein coding and 43 structural RNAs. Treatment of the RNA with TEX prior to sequencing removes processed, fragmented and degraded RNA molecules with a 5' monophosphate from the total RNA. By selective digestion of RNA with 5' monophosphates, native 5' ends carrying a triphosphate were enriched. This enables the exact determination of TSSs at single nucleotide resolution as previously demonstrated for the human pathogens *Helicobacter pylori *[24] and *Chlamydia trachomatis *[21], the cyanobacterium *Synechocystis *[20], the archaeon *Methanosarcina mazei *[25], and the Gram-positive bacterium *Bacillus subtilis *[26].

**Figure 2:**
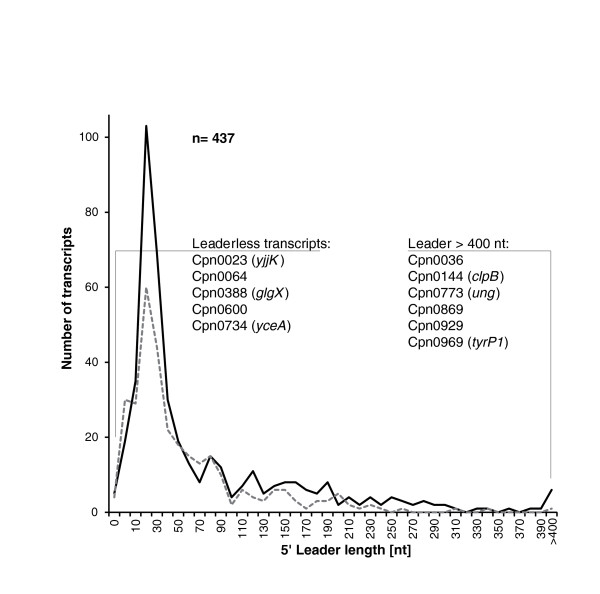
**5' leader length distribution and leaderless mRNAs**. The graph shows the distribution of the distances between the TSS and the translation start site of 437 annotated protein coding genes for which a distinct TSS could be assigned. Five leaderless genes could be identified where the TSS corresponds to the annotated translation start site. Six genes have very long leader sequences >400 nucleotides in length. For comparison, leader lengths of 320 *Ctr *genes are included (dashed grey line) from a previous study [21]. Nt, nucleotides.

In total, 531 primary TSSs and 34 secondary TSSs, located downstream of primary TSSs, could be identified by manual inspection of the sequencing data (listed in Table S2 in Additional file 2). Based on the TSS map, we calculated the length of 5' leader sequences for the 437 mRNAs with assigned TSSs. Leader sequences of the majority of mRNAs varied between 10 and 50 nucleotides in length. Leaders longer than 100 nucleotides were found for 111 mRNAs; Cpn0036, *clpB*, *ung*, Cpn0869, Cpn0929, and *tyrP1 *have leaders of even more than 400 nucleotides. On the contrary, Cpn0064, *yjjK*, *glgX*, Cpn0600, and *yceA *are transcribed as leaderless mRNAs whose TSSs and translation start sites are identical. A comparison of the leader lengths between *Cpn *and *Ctr *shows a very similar size distribution between the two species (Figure 2). Two novel protein coding genes that were missing in the annotation have been identified: Cpn0600.1 is a homologue of *Cpn *strain AR39 gene CP0147; and Cpn0655.1 is located antisense to Cpn0955 and contains an ORF of 72 amino acids.

The analysis of mRNA leader lengths revealed ten genes that have to be re-annotated because their TSS is located downstream of the annotated translation start site (Table S3 in Additional file 1). Alternative shorter ORFs that are consistent with the TSS are present in all of these genes. For example, the heat shock transcriptional regulator HrcA is encoded as the first gene of the *dnaK *operon and starts 8 bp downstream of the annotated coding sequence. An in-frame start codon is downstream of the annotated TSS and consequently the protein has a 12 amino acid shorter amino terminus than previously predicted.

**Figure 3:**
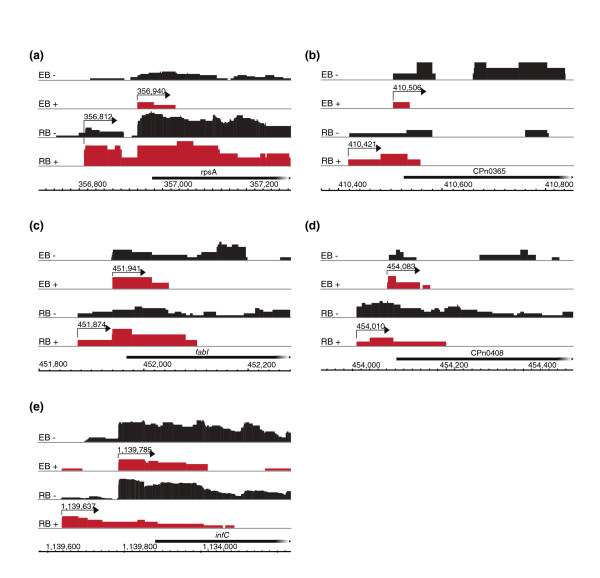
**Genes with alternative transcriptional start sites in *C. pneumonia *elementary bodies and reticulate bodies**. Visualization of sequence reads for five genes that show alternative TSSs in EBs and RBs. Black graphs represent reads from untreated libraries of EBs (EB-) and RBs (RB-), respectively, and red graphs represent reads obtained from TEX-treated libraries of EBs (EB+) and RBs (RB+), respectively. Arrows depict TSSs with the genomic coordinates of the TSS above. The TSS is located downstream in EBs, which results in a shorter 5' leader sequence.

Several genes have been described to have tandem promoters because two or more potential TSSs have been mapped upstream of the gene. These are *Chlamydia trachomatis tuf *[27], the rRNA gene [28], and *ompA *[29]. In *Cpn*, however, the *tuf *gene is co-transcribed as part of an operon and has no TSS upstream of the gene start. For the rRNA gene, a single TSS could be identified and a processing site at position 1,000,490, which was previously reported to be a TSS in *Chlamydia muridarum *[30]. Tandem promoters with alternative TSSs were identified for 18 genes (Table S2 in Additional file 2). Interestingly, among these were genes with tandem promoters that are differentially used for transcription in EBs and RBs, such as *rpsA*, CPn0365, *fabI*, CPn0408 and *infC *(Figure 3). The sequencing read distribution of the enriched cDNA libraries of these genes demonstrated TSSs in EBs downstream of the TSSs in RBs, resulting in a shorter leader sequence of the mRNAs in EBs. This developmental use of alternative promoters could influence mRNA stability or structure or translational activity. Use of stage-specific alternative TSSs gives insights into possible mechanisms of stage-specific gene regulation. The presence of developmental stage-specific promoters has been demonstrated previously for the *Ctr *cryptic plasmid gene pL2-02 [21,31]. Alternative promoters could be detected by stage-specific transcription factors resulting in different lengths of mRNA leader sequences and the presence or absence of regulatory elements.

All 21 members of the important group of polymorphic outer membrane proteins (Pmp) were found to be expressed. The detailed list of TSSs in Table S2 in Additional file 2 shows that an internal TSS was found to be located inside the annotated *pmp3.2 *gene, resulting in a transcript of 1.5 kb that contains an ORF of 454 amino acids in-frame to the annotated protein of 746 amino acids. Furthermore, internal TSSs were present in *pmp5.1*, *pmp10.1*, and *pmp17.1*. The *ompA *gene encodes the major outer membrane protein of *Chlamydia*, which constitutes more than 60% of the total outer membrane protein content [32]. With a total of 3,749 reads *ompA *was the second most abundant protein coding gene after the 'cysteine-rich outer membrane protein' coding gene *omcB *(9,009 reads) in terms of read numbers per gene. The *C. trachomatis ompA *gene was first described to have two tandem promoters that give rise to two transcripts that are differentially expressed during the life cycle [33]. Douglas and Hatch [34] could show that *in vitro *transcription occurs only from the upstream TSS (Figure S4A in Additional file 1; position 60,074) and the shorter transcript is a fragment of the longer primary transcript. The sequencing read distribution of our previous dRNA-seq analysis in *C. trachomatis *[21] confirms this assumption, since only one major primary TSS was found upstream of *ompA *at position 60,074 (P2; Figure S4A in Additional file 1). A minor TSS represented by only one cDNA sequence is located 26 bp upstream (P1; Figure S4A in Additional file 1). The -25 position (at 59,852) seems to be a processing site because a number of transcripts start at this position in the untreated library but none do so in the TEX-treated libraries. Interestingly, in *Cpn *the *ompA *gene seems to have three distinct TSSs upstream of the coding sequence in the TEX-treated libraries (P1 to P3; Figure S4B in Additional file 1), all of them harboring a σ^66 ^promoter sequence (Figure S4C in Additional file 1). Two minor TSSs are located at -266 and -254 (positions 779,949 and 779,961, respectively) and one major TSS is found at -165 (position 780,050). Interestingly, only P2 is conserved between *Ctr *and *Cpn*. The major TSS P3 is only present in *Cpn *even though the -10 and -35 boxes are conserved between *Cpn *and *Ctr *(Figure S4D in Additional file 1). For all *ompA *RNA species more sequence reads were obtained from the RB than from the EB libraries, indicating increased expression of OmpA in RBs, as previously described [33].

### Annotation of operon structure

The combined analysis of cDNA libraries derived from total RNA and RNA enriched for TSSs allowed us to analyze the operon structure of the *Cpn *genome. For example, two of the operons that encode genes of the type three secretion system (T3SS) [35] were expressed and sequence reads were present for the entire operons in the untreated cDNA libraries (black graphs of Figure S5 in Additional file 1). In contrast, sequence reads of the enriched libraries define two distinct TSSs in the first operon of five genes (red graphs in Figure S5A in Additional file 1); one TSS is located upstream of the *yscU *gene and the other internal TSS is located upstream of *lcrE *and inside the coding sequence of *lcrD*. This operon is therefore likely transcribed as one long transcript comprising all five genes and a shorter transcript derived from the internal promoter that encodes the three genes *lcrE*, *sycE *and *MalQ*. The other operon encodes six genes and has only one distinct TSS (red graphs in Figure S5B in Additional file 1).

We investigated all 799 adjacent gene pairs identified in the genome of *Cpn *using a similar approach and found 246 polycistronic transcripts from a total of 752 genes organized in pairs of 2 to 25 ORFs each (Table S4 in Additional file 1). In summary, a distinct TSS or an affiliation to a polycistronic transcriptional unit with a distinct TSS could be precisely assigned to 861 out of 1,074 protein coding genes (80%) in the *Cpn *transcriptome (Table S2 in Additional file 2).

Several algorithms for operon prediction have been published in recent years. The present data set of operons of *Cpn *was compared with published operon predictions available at MicrobesOnline [36] and the DOOR database [37]. Of the 799 pairs of adjacent genes, 721 pairs (90.2%) could be classified as either co-transcribed or individually transcribed. The remaining 78 pairs could not be classified since sequence read numbers were too low and discrimination between co-transcription or individual transcription was thus not possible. The comparison with theoretical operon prediction algorithms reveals that 78.6% (DOOR) and 81.1% (MicrobesOnline) of the predictions coincide with the experimental data. Consequently, the consistency of operon predictions and experimental data is of the same magnitude as found for other bacteria like *H. pylori *[24].

### Identification of *cis- *and *trans-*encoded small RNAs

**Figure 4:**
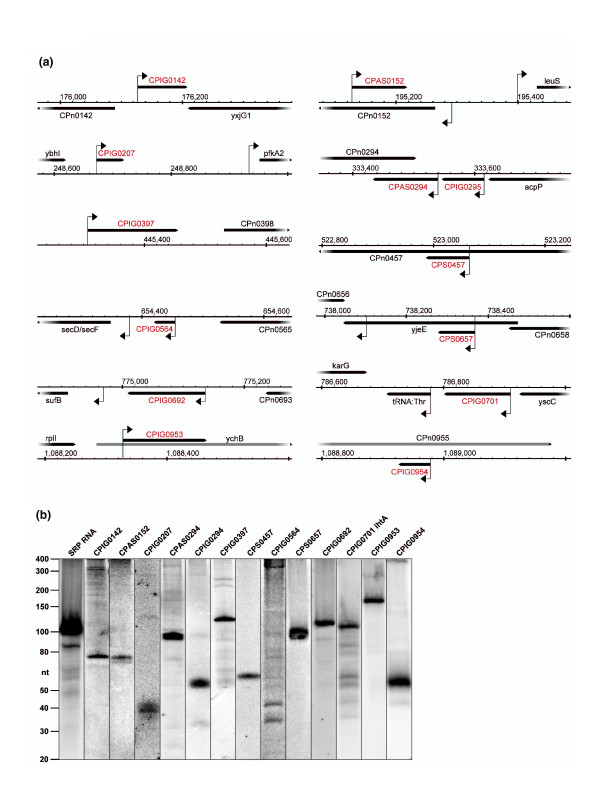
**Novel non-coding sRNAs of *C. pneumonia***. **(a) **Short novel transcripts lacking an ORF were identified in intergenic regions, antisense or sense to annotated protein coding genes. The x-axis represents genomic coordinates with annotated genes on the plus (above) and minus strand (below). Newly assigned names of sRNAs are colored in red. Arrows represent TSSs of sRNAs and surrounding genes as determined from deep-sequencing data. The genes *ychB *(CPn0954) and CPn0955 seem to be wrongly annotated and are therefore depicted in grey. **(b) **Northern blot analysis of the small transcripts verifies the theoretical sizes calculated from the deep sequencing data. The housekeeping signal recognition particle RNA (SRP RNA) was used as a positive control for size verification. CPIG0701 is a homologue of the only previously identified sRNA in *C. trachomatis*.

Numerous small transcripts lacking an ORF could be identified in intergenic regions, antisense or even sense to protein coding genes. In total, 75 TSSs (listed in Table S2 in Additional file 2) were indicative of putative sRNAs. These comprise 20 putative *trans-*acting sRNAs encoded in intergenic regions, 47 putative *cis-*encoded antisense sRNAs and 8 sRNA candidates encoded sense to annotated ORFs. The 54 most promising candidates for novel sRNAs were analyzed by Northern hybridization to test for the presence of a distinct band of the corresponding size. Thirteen of these sRNA candidates were positively validated (Figure 4a,b). Nine novel *trans*-acting sRNAs are transcribed from intergenic regions, two *cis*-acting antisense sRNAs are transcribed from annotated protein coding genes, and three sRNAs are encoded inside the coding regions (Figure 4a). The validated novel sRNAs are numbered according to the protein coding gene encoded upstream, antisense or sense to the sRNA, respectively. A comparison to recently discovered sRNAs in *Ctr *[21,22] revealed only three sRNAs: CPIG0564 (homologue to CTIG449), CPIG0692 (homologue to CTIG684), and CPIG0701 (IhtA) are conserved in *Cpn *and *Ctr*. Most of the remaining novel sRNAs are encoded adjacent to genes that are not conserved in *Ctr*. All predicted house-keeping RNAs were identified, including tRNAs, 5S, 16S and 23S rRNAs, signal recognition particle RNA (SRP RNA, 4.5S RNA; Figure 4b), trans messenger RNA (tmRNA) and RNaseP RNA (M1 RNA). Furthermore, the homologue of the previously described sRNA *IhtA *in *C. trachomatis *[23] could be detected (CPIG0701; Figure 4b). Twenty-six of the tested sRNA candidates gave no signal in Northern hybridization, probably due to weak expression or insufficient probe binding, and 14 candidates gave a signal that did not correspond to the theoretical size obtained from the sequencing data.

**Figure 5:**
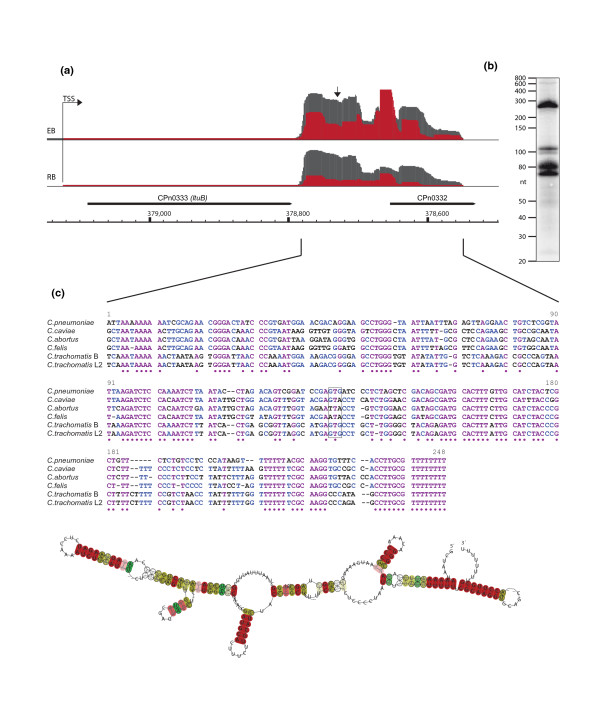
**An abundant putative small RNA encoded in Cpn0332**. **(a) **Visualization of sequencing reads of the CPn0332 locus. Grey bars represent untreated total RNA libraries and red bars represent libraries of RNA treated with TEX and thus are enriched for primary transcripts. The upper line shows sequence reads obtained from EBs and the lower those from RBs. Black bars show annotated genes and coordinates of the genome. A highly abundant transcript is co-transcribed with *ltuB*. It ends 18 nucleotides upstream of the annotated stop codon of CPn0332 and lacks an alternative ORF. **(b) **Northern blot analysis of the abundant transcript reveals several bands ranging from approximately 70 to 250 nucleotides. The probe binding site is marked by a vertical arrow in (b). **(c) **Alignment of the locus downstream of *ltuB *shows homology between all available *Chlamydia *genomes and the two *C. trachomatis *strains B and L2. The boxed area marks the start codon of the annotated gene CPn0332, which is not conserved. **(d) **The RNA consensus secondary structure estimated from the RNA sequence alignment in (c). The colors indicate the base pair probabilities. The accumulation of red collared base pairs indicates the high reliability of the estimated consensus structure. The free energy of the thermodynamic ensemble is -106.37 kcal/mol.

CPn0332 is one of the most abundant transcripts, with a total of 40,170 sequence reads in the four cDNA libraries. The transcript is located downstream of *ltuB*, which encodes the 'late transcription unit B' gene, lacks its own TSS and is co-transcribed with *ltuB *(Figure 5a). It was previously described for *C. trachomatis *as an accumulating fragment of the *ltuB *transcript [17]. The transcript is 18 nucleotides shorter than the annotated gene CPn0332 and no alternative ORF is present. Northern blot analysis reveals a full-length RNA species of approximately 250 nucleotides in length, which fits well with the theoretical size of 238 nucleotides. Several smaller fragments could be detected by the probe, ranging from 70 to 110 nucleotides in length (Figure 5b). Homologues of the full-length sequence were present in all available *Chlamydia *genomes (Figure 5c) and we previously identified a very similar transcript in *C. trachomatis *[21]. It contains several highly conserved regions and a conserved intrinsic terminator stem-loop followed by a poly-T stretch. The start codon of the annotated ORF is not conserved among all *Chlamydia*, which supports our findings of a non-coding RNA encoding locus instead of a protein coding gene.

Miura *et al*. [16] searched for transcripts that are expressed at the late stage of the infection cycle and identified putative σ^28 ^promoters upstream of *ltub *and the annotated ORF CPn0332. According to the identified TSS, the putative σ^28 ^promoter postulated upstream of ORF CPn0332 is located inside the transcript. In many proteobacteria, 6S RNA was identified to be an abundant non-coding RNA that globally regulates transcription during growth phases by inhibition of standard sigma factor RNA polymerase [38] and thereby enhance alternative sigma factor activity. 6S RNA mimics an open promoter complex and a part of this RNA resembles a DNA promoter sequence [39]. We tested whether the σ^28 ^binding site is functional, which would result in binding of σ^28 ^RNA polymerase to this RNA. We therefore tested by gradient fractionation whether CPn0332 RNA co-sediments with σ^28 ^RNA polymerase. However, an association of RNA CPn0332 with σ^28 ^could not be confirmed since the CPn0332 RNA and σ^28 ^were found in different fractions (Figure S6 in Additional file 1). Furthermore, an association of CPn0332 with ribosomes can be excluded since the RNA does not co-sediment with ribosomal RNAs (Figure S6 in Additional file 1).

Although an association of CPn0332 RNA with σ^28 ^RNA polymerase and ribosomes could be excluded, RNA polymerase itself and other σ factors could be tested as soon as antibodies for these proteins are available. Also, an identification of binding partners by aptamer-tagging technology could shed light on the biological role of this sRNA [40].

### Differences in the EB and RB transcriptomes

Previous studies on gene expression during the course of the *Cpn *developmental cycle were based on RNA isolation from infected host cells without further purification of the bacteria. Since the developmental cycle of *Chlamydia *becomes increasingly asynchronous with time, this results in a mixture of EB, RB, and intermediate forms at the late time points of infection. Here we were able to isolate EBs and RBs by differential gradient centrifugation to obtain total RNA from the two distinct life cycle forms.

For analysis of differential gene expression 1,012 genes were considered. According to the settings applied (threshold of ≥20 sequence reads per gene, twofold difference in abundance, *P *≤ 0.05), 288 genes were classified as differentially expressed (Table S6 in Additional file 3). Of these, 83 previously annotated genes and eight novel putative sRNA genes were more abundant in EBs and 192 annotated genes as well as five putative sRNA genes were more abundant in RBs. Interestingly, we found 68% and 24% of these enriched genes to be hypothetical proteins of unknown function in EBs and RBs, respectively. Gene families more abundant in RBs comprise most house-keeping genes, that is, genes involved in DNA and RNA synthesis, cell division, energy metabolism as well as the polymorphic outer membrane proteins. Among the few known transcripts more abundant in EBs is the *ltuA *(late transcription unit A) gene. The *ltuB *RNA is only 1.6-fold more abundant in EBs than in RBs. Since this gene is transcribed late in the developmental cycle and the transcripts are very abundant, this RNA seems to accumulate in EBs. A comparison to differentially expressed genes of *Ctr *reveals that half of the hypothetical proteins enriched in *Cpn *EBs are only poorly conserved in *Ctr *or have no homologous gene at all. Among the genes enriched in *Cpn *EBs are 14 encoding putative inclusion membrane proteins containing the IncA domain (Pfam PF04156). These include CPn0585, which has been demonstrated to be localized in the inclusion membrane and interact with host cell Rab-GTPases [20], as well as CPn1027 [41] and CPn0308 [40], which have also been shown to be localized in the inclusion membrane.

A comparison of all differentially expressed genes with microarray data of an infection time course by Mäurer *et al*. [11] showed that 83% of the genes we found more abundant in EBs have their expression maximum at 6 or 72 hours post-infection. At these time points EBs are predominant. In contrast, 74% of the genes enriched in RBs have their expression maxima at intermediate time points 12 to 60 hours post-infection in which RBs are predominant. This indicates a good concordance between both approaches. These results correlate well with a comparison of differential gene expression of *Ctr *EBs and RBs between dRNA-seq and microarray data sets in our previous study [21].

All 14 genes encoding IncA domain-containing proteins were found to have their maximum expression at 6 h or 72 h in the microarray data set [11]. Since EBs lack transcriptional activity, the mRNAs accumulated in EBs could be stored for immediate expression upon conversion into RBs. Thus, the IncA domain-containing proteins could be among the first effectors secreted into the host cell to be incorporated into the inclusion membrane. Furthermore, it has been discussed that 'carry-over' mRNA that is abundant in EBs does not lead to protein synthesis early in the infection cycle but to rapid degradation [11,42]. The mechanisms of distinguishing pre-stored mRNA for immediate translation and carry-over mRNA that is degraded are unclear. Analysis of differentially expressed genes showed that eight novel sRNA transcripts were found to be more abundant in EBs. The enrichment of putative sRNAs in EBs could indicate a mechanism of post-transcriptional gene regulation and mRNA degradation upon reactivation of translation early in the infection cycle. Thus, the carry-over mRNAs could be targeted by the sRNAs stored in EBs and translation could thereby be sequestered.

Genes more abundant in EBs are mostly of unknown function and the mechanism of EB to RB conversion is poorly understood. Besides the protein coding genes, non-coding RNAs like CPn0332 could be involved in the control of the developmental cycle, since it is 2.4-fold upregulated in EBs compared to RBs.

### Analysis of *C. pneumoniae *promoters

**Figure 6:**
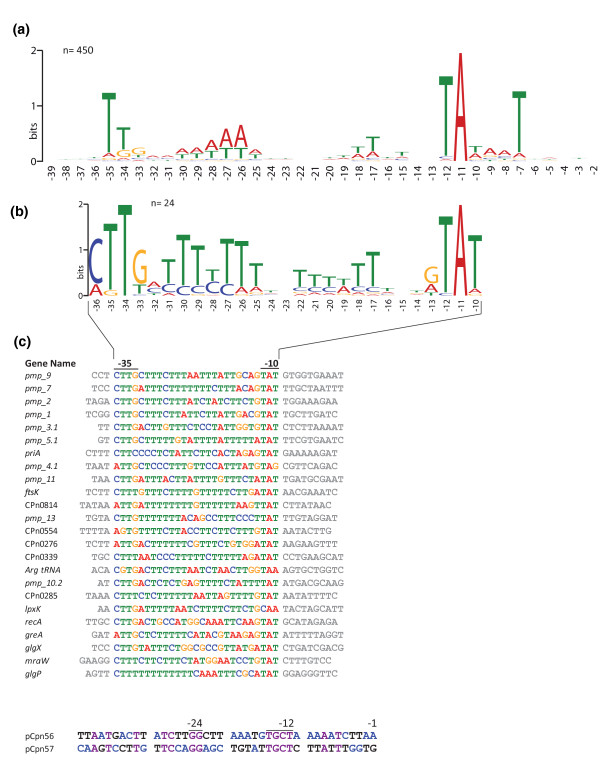
**Promoter motifs of *C. pneumonia***. Sequences upstream of 531 TSSs were extracted (positions -1 to -40) and common sequence motifs were searched using MEME software [45]. The coordinates give positions relative to TSSs. **(a) **The most prominent sequence alignment results from 450 out of 531 promoter sequences that were aligned. The motif resembles previously described σ^66 ^promoter sequences and has the typical *E. coli *σ^70 ^consensus sequences TATAAT at the -10 box. The -35 box is less conserved but is also similar to the *E. coli *consensus sequence TTGACA. For the promoter alignment a sequence shift of ±1 nucleotides from the TSS was permitted. **(b) **The second most prominent motif is derived from an alignment of 24 genes, of which 10 belong to the polymorphic outer membrane protein family (Pmp). The sequence contains well conserved -35 and -10 boxes and a T-rich spacer in between. **(c) **A search for the minimum conserved σ^54 ^consensus sequence GG-N_9-11_-GC returned only two putative σ^54 ^promoters that belong to sRNA candidates. No such sequence motif could be found in promoter regions of protein coding genes.

Bacterial RNA polymerase contains alternative σ factors that bind to conserved nucleotide sequences upstream of the TSS to initiate transcription. Based on manual determination of single TSSs and biocomputational analysis of promoter sequences, a few promoter sequence motifs have been identified so far [17,43,44], most of them for *Ctr*. The data set generated in this study offered the unique opportunity to precisely define positions upstream of the TSSs and thus compare potential promoter consensus sequences. We started by extracting the sequences 40 bp upstream of the 531 determined primary TSSs and analyzed them for common motifs. The genome-wide promoter analysis based on pairwise local alignments of all 531 promoter sequences indicates only a very weak conservation structure. However, a weak clustering of the promoter sequences of the PMP gene family could be observed (data not shown). Using MEME [45] a common motif could be found that resembles the *E. coli *σ^70 ^consensus sequence in 450 out of 531 promoter regions (Figure 6a). The determined -35 box consensus motif TTGA is shorter than the *E. coli *consensus sequence (TTGACA) but the -10 box resembles the *E. coli *sequence (TATAAT) whereas only TANNNT is highly conserved (Figure 6a). In addition, between the -10 and the -35 box are two A/T rich stretches around positions -17 and -26 in *Cpn*. These sequences (Figure 6a) resemble the putative consensus promoter sequence of *Cpn *σ^66 ^RNA polymerase [46,47].

An additional promoter motif was detected for 24 genes, of which 10 genes belong to the polymorphic outer membrane protein family (Pmp) (Figure 6b). These promoters share the motif CTTG at the -35 region and GTAT at the -10 box with long T-rich regions in between. The MEME algorithm cannot be used to find common promoter regions with differences in the spacer regions between the -10 and -35 box. To overcome this limitation, a search for common motifs was done for the -35 and -10 regions separately. The predominant motifs found (Figure S7 in Additional file 1) resemble the σ^66 ^consensus sequence shown in Figure 6a. This result indicates that the spacer region seems to be of constant length.

Several predicted and validated promoter motifs have previously been reported in *Chlamydia*. The most conserved bacterial promoter sequence is the σ^54 ^promoter with the consensus sequence TGGCAC-N_5_-TTGC [48]. Studholme and Buck [49] identified a putative σ^54 ^promoter sequence upstream of the *Cpn *gene AAD18864 (CPn0725) located at positions 810,800 to 810,815. This site is entirely located inside the transcript of CPn0725 and overlaps with the coding sequence. A promoter site at this position is thus unlikely. Mathews and Timms [43] searched for putative σ^54 ^promoter consensus sequences in the *Chlamydia *genomes and identified a further putative σ^54 ^binding site upstream of CPn0693. The sequencing data and also the Microbes Online operon prediction [50] suggested a co-transcription of CPn0693 and CPn0694 from a TSS upstream of CPn0694, arguing against a potential σ^54 ^promoter upstream of CPn0693. Of the nine putative σ^54 ^promoters identified in *Ctr*, we could confirm none in *Cpn *because the homologous gene either is lacking or has no putative σ^54 ^promoter in the region upstream of the TSS.

To further elucidate the presence of σ^54 ^promoters, the 531 extracted promoter sequences (positions -1 to -40 relative to the TSS) were tested for the least conserved σ^54 ^core sequence GG-N_9-11_-GC of the -24 and -12 boxes, respectively. In this data set of 531 promoter sequences no putative σ^54 ^promoter sequence could be identified for annotated protein coding genes. However, two putative σ^54 ^promoters were identified upstream of the novel sRNA candidates pCPn56 and pCPn57 (Figure 6c), which share sequence homology only at the -12 and -24 promoter boxes, respectively.

The third sigma factor identified in *Chlamydia *so far is σ^28 ^and was shown to be expressed at the late stage of infection. Yu *et al*. [51] identified putative σ^28^-regulated genes in *C. trachomatis *by an *in silico *prediction algorithm. Using an *in vitro *transcription assay they could verify five genes, *tlyC1*, *bioY*, *dnaK*, *tsp *and *pgk*, to be controlled by σ^28^. Two of these genes (*tsp *and *pgk*) are expressed in *Cpn *from their own TSSs under the control of a promoter that resembles the predicted *C. trachomatis *consensus promoter. The *tsp *TSS supports the predicted promoter sequence, but there is weak sequence homology of the promoter region of *pgk *between *Ctr *and *Cpn*. The genes *tlyC1 *and *dnaK *are co-transcribed as part of a polycistronic transcript and *bioY *(probable biotin synthase) has no homologous gene in *Cpn*.

Several studies have characterized temporal gene expression during the developmental cycle of *Chlamydia *using microarrays [11,16,42,52]. These studies identified a cluster of genes that are expressed at a late stage of infection corresponding to the stage prior to conversion of RBs to EBs. This set of genes includes *hctB*, which was shown to be recognized by σ^28 ^[18]. The *Cpn hctB *promoter contains the extended σ^28 ^consensus sequence TNAAG-N_14_-GCCGATA derived from several γ-proteobacteria sequences [53] with a spacer of 15 nucleotides. In the set of 531 promoter sequences no further sequence was found that resembles the described σ^28 ^promoter sequence of *hctB *TNAAG-N_15_-GCC. A search for the same motif but using a variable spacer length of 12 to 16 nucleotides in length returned only *tsp *(tail specific protease; CPn0555), which exactly matches the consensus sequence with a spacer of 14 nucleotides.

A search for the σ^28 ^consensus sequence of the -35 box (TNAAG) returned 22 more sequences. None of these sequences contains the minimum -10 box sequence GCC or CGA, which was shown to be the preferred -10 sequence in a mutational analysis of the *hctB *promoter by Yu *et al*. [44]. Three of the late genes have been predicted by Miura *et al*. [16] to have σ^28 ^promoter sequences based on homology to the known *hctB *promoter -35 box AAAGTTT. The TSS data set argues against the existence of these promoter sites. For example, the predicted promoter of *adk *is located inside the transcript and we could not identify an alternative TSS upstream. CPn0332 is co-transcribed with CPn0333 and does not have its own promoter and the predicted *ltuB *-35 box is located at position -26 upstream of the TSS. Furthermore, these authors showed a homologous region upstream of the genes CPn0331, *omcA*, CPn0678 and *hctA*. Since this region starts at different distances relative to the corresponding TSSs of these genes (CPn0331, -86; *omcA*, -33; CPn0678, -80; *hctA*, -65), it is unlikely that these sequences are part of a common promoter.

The global analysis of promoters shows that most genes in *Cpn *are controlled by the standard σ^66 ^promoter, which has a common motif that is less conserved than in other bacteria. Since no common promoter motif could be identified for genes overrepresented in EBs and RBs, it is likely that differential expression of these subsets of genes is not accomplished by the use of alternative σ factors. Other sequence motifs, such as transcription factor binding sites, may be present and act as *cis*-regulatory elements to control alternative gene expression. In addition, since the *Cpn *genome is densely packed and intergenic regions are short, gene regulation could be effected by other mechanisms, such as through sRNAs or antisense RNAs, which have been identified in this study.

## Conclusions

We successfully applied dRNA-seq to analyze differential gene expression in purified EBs and RBs of *Cpn*. Our results provide new insights into the transcriptional organization, gene structure and promoter motifs of *Cpn*. A common promoter motif could be identified for the standard σ^66 ^factor, whereas a conserved promoter motif for the two alternative sigma factors could not be identified. Gene regulation seems to be controlled by a multitude of non-coding RNAs that were identified and in part experimentally confirmed. These results are the basis for further investigation of chlamydial gene regulation using heterologous or *in vitro *systems.

## Materials and methods

### Infection and isolation of vacterial RNA

Hep-2 (ATCC CCL-23) cells were cultured in DMEM containing 10% fetal bovine serum and infected with *Cpn *strain CWL-029 (ATCC VR1310) with a multiplicity of infection (MOI) of 5 for 24 and 72 hours. *Cpn *containing cells were collected by scraping, pooled and disrupted with glass beads. All steps were performed on ice or at 4°C. *Chlamydia *were isolated by differential centrifugation followed by density gradient centrifugation in a discontinuous sucrose density step gradient. Cells were disrupted and crude *Chlamydia *pellets were obtained as described before for *C. trachomatis *[21]. The bacterial pellet was resuspended in 1 ml of ice cold SPG buffer without using a syringe to avoid mechanical disruption of RBs. Then, *Cpn *suspension was layered on top of the sucrose step gradient followed by centrifugation for 60 minutes at 4°C and 30,000 rcf in a swing out rotor. After centrifugation, EBs and RBs were present as distinct bands at the interphases. EBs and RBs were carefully collected by capillary pipettes and washed in SPG buffer. The purity of the pellets was estimated by electron microscopy.

Pelleted bacteria were resuspended in Trizol (Invitrogen, Darmstadt, Germany) and RNA was isolated according to the manufacturer's protocol with addition of an initial mechanical disruption in a homogenizer (FastPrep, MP Biomedicals, Illkirch, France) using 1.5 ml Lysing Matrix B tubes for 4 bursts of 25 s each at maximum speed and dry ice cooling. Contaminating DNA was digested by DNAseI (Fermentas, St. Leon-Rot, Germany; 0.5 U/mg RNA, 30 minutes, 37°C) in the presence of RNAse inhibitor (RiboLock, Fermentas; 0.1 U/μl) followed by isolation of RNA by phenol/chloroform/isoamylalcohol and precipitation of RNA by 2.5 volumes of ethanol containing 0.1 M sodium acetate. The absence of DNA was controlled by PCR using primers to amplify genomic DNA of the *ompA *gene. RNA quality was determined on a Bioanalyzer 2100 using RNA 6000 Nano kit (Agilent, Böblingen, Germany). Absence of 18S and 28S eukaryotic ribosomal RNA peaks supported the purity of the bacteria preparation.

### Preparation of cDNA and sequencing

Primary transcripts of total RNA were enriched by selective degradation of RNAs containing a 5' mono-phosphate (5'P) by treatment with 5' P-dependent TEX (Epicentre #TER51020, Madison, WI, USA). Primary bacterial transcripts (most mRNAs and sRNAs) are protected from exonucleolytic degradation by their tri-phosphate (5'PPP) RNA ends. Total RNA was freed of residual genomic DNA by treatment with 1U DNase I per microgram of RNA for 30 minutes at 37°C. For depletion of processed transcripts, equal amounts of *Chlamydia *RNA were incubated with TEX or in buffer alone for 60 minutes at 30°C. One unit of TEX was used per 1 μg total RNA. Following organic extraction (25:24:1 v/v phenol/chloroform/isopropanol), RNA was precipitated overnight with 2.5 volumes of an ethanol/0.1M sodium acetate (pH 6.5) mixture, and treated with 1 unit Tobacco Acid Pyrophosphatase (Epicentre, Madison, WI, USA) for 1 hour at 37°C to generate 5'-mono-phosphates for linker ligation, and again purified by organic extraction and precipitation as above.

cDNA cloning and pyrosequencing were performed as described before [54] but omitting size fractionation of RNA prior to cDNA synthesis. Equal amounts of total RNA were used for the generation of all cDNA libraries. For linker ligation, RNA was treated with TAP to generate 5'-mono-phosphates. After addition of specific 5' linkers with unique tags for each library and poly-A-tailing, the RNA was converted into a cDNA library. Two sets of four cDNA libraries each were generated in total: total RNA and total RNA enriched for primary transcripts from EBs and RBs, respectively. The first sequencing run was performed using Roche FLX chemistry. A second set of cDNA libraries was optimized for the sequencing conditions of the Roche Titanium chemistry. Sequence reads derived from both sequence runs were pooled for each library (Table S1 in Additional file 1). Sequencing raw data can be found and downloaded at the Gene Expression Omnibus (GEO) database under the accession number [GSE24999] [55].

### Analysis of sequences and statistics

From the multiplex sequencing runs the sequence reads were sorted by their specific four base barcodes, which were added during 5' linker ligation during cDNA synthesis. Clipping of 5' linker and poly-A tails was performed and all cDNA sequence reads ≥18 nucleotides were considered for BLAST (Basic Local Alignment and Search Tool) search. The sequences were aligned to the *Cpn *CWL-029 genome (NC_000922) using WU-BLAST 2.0 [56] with the following parameters: -B = 1 -V = 1 -m = 1 -n = -3 -Q = 3 -R = 3 -gspmax = 1 -hspmax = 1 -mformat = 2 -e = 0.0001.

For visualization of BLAST hit locations, graph files were calculated and loaded into the Integrated Genome Browser version 4.56 [57] as previously described [58]. From the resulting BLAST data two graphs were calculated for every library, one for the sense and one for the antisense strand. Each graph represents the number of cDNA reads obtained from the sequencing for every nucleotide position.

To predict the consensus secondary structure of a set of RNA sequences the RNAalifold web server [59] was used with default settings. For the promoter analysis promoter sequences (-1 to -40 upstream of TSSs) have been extracted from 531 genes with annotated TSSs. These sequences were compared by calculating all against all local pairwise alignments using the Smith-Waterman algorithm as implemented in Biostrings (R package version 2.18.4) using R version 2.12.2 [60]. Due to the strong compositional bias in the promoter sequences, a composition adjusted scoring matrix based on the Felsenstein model [61] was calculated and linear gap costs of -7 were used (Figure S8 in Additional file 1). For all resulting alignment scores empirical *P*-values were calculated based on background scores derived from pairwise alignments of randomly sampled sequences with the same base composition and length.

For the detection of differentially expressed transcripts all genes with at least 10 sequence reads in total and a maximal read count lower than 1,000 were considered. To account for the different conditions of TEX-treated and untreated samples the Mantel-Haenszel test was used, as implemented in the R function mantelhaen.test. This statistical test of conditional independence within strata extends Fisher's exact test to account for additional experimental effects [62]. The resulting *P*-values were multiple testing corrected by the Benjamini-Hochberg procedure [63].

### Northern blot analysis of sRNAs

Total RNA (50 μg) was diluted with an equal amount of 2× RNA gel loading buffer, denatured for 5 minutes at 95°C and quick chilled on ice. The RNA was then separated on a denaturing 8% polyacrylamide gel (1× TBE buffer, 8 M urea) in 1× TBE. For size detection, the Decade Markers (10 to 150 nucleotides; Ambion, Foster City, CA, USA) and the RiboRuler low range marker (100 to 1,000 nucleotides; Fermentas) were used according to the manufacturers' protocols. The transfer of RNA to a positively charged nylon membrane was carried out by wet blot transfer in 0.5× TBE buffer for 3 hours at 4°C. After blotting the RNA was UV cross-linked to the membrane by exposure of 120 mJ. Prehybridization of the membrane was carried out in RapidHyb buffer at 42°C for at least 1 hour. The DNA probe (10 pmol) was labeled with γ-^32^P-ATP and T4-Polynucleotide kinase (PNK) in the supplied buffer for 1 hour at 37°C. After heat inactivation of the PNK for 3 minutes at 95°C, the labeled probe was purified by a Sephadex-25 gel filtration column according to the manufacturer's protocol. For hybridization, the radioactive labeled probes were directly added to the prehybridization buffer and incubated for 16 hours at 42°C.

Following incubation, the membranes were washed in prewarmed washing buffer (2× SSC, 0.1% SDS) at 42°C. Then the membranes were wrapped in plastic film and exposed to phospho-storage plates (FujiFilm). The screens were read by a Typhoon scanner (Molecular Devices) and results were visualized by LabImager image analysis software.

## Abbreviations

bp: base pair; *Cpn*: *Chlamydia pneumoniae*; *Ctr*: *Chlamydia trachomatis*; dRNA-seq: differential RNA sequencing approach; EB: elementary body; ORF: open reading frame; RB: reticulate body; sRNA: small non-coding RNA; TEX: terminator exonuclease; TSS: transcription start site.

## Competing interests

The authors declare that they have no competing interests.

## Authors' information

MA, CMS, JV and TR designed the study, RR carried out the deep sequencing, CMS did the raw data processing, MTD and TM carried out the statistical analysis of gene expression, MA did the remaining experiments and data analysis, MA and TR wrote the manuscript. All authors read and approved the final manuscript.

## Supplementary Material

Additional file 1 **Supplemental figures, tables and methods**. Click here for file

Additional file 2 **Table S2 - transcription start sites and sequencing read numbers of *Cpn *genes**. A table listing the *Cpn *genome annotation including all identified TSS, novel transcripts and sequence read numbers for all transcripts. Click here for file

Additional file 3 **Table S6 - list of relative gene expression in EB and RB**. A table listing the 288 genes more abundant in EB or RB. Click here for file
