# Supplementary material for: The transcriptional landscape of Chlamydia pneumoniae
Source: Genome Biol. 2011 Oct 11;12(10):R98. doi: 10.1186/gb-2011-12-10-r98 (PMC3333780; doi:10.1186/gb-2011-12-10-r98)
Supplement: Additional file 1 — Supplemental figures, tables and methods. [file gb-2011-12-10-r98-S1.PDF]

The Transcriptional Landscape of *Chlamydia pneumoniae*

Marco Albrecht, Cynthia M. Sharma, Marcus Dittrich, Tobias Müller, Richard Reinhardt, Jörg Vogel, Thomas Rudel

Supplemental Data

Supplemental Figures

Supplemental Tables

Supplemental Methods

Figure S1

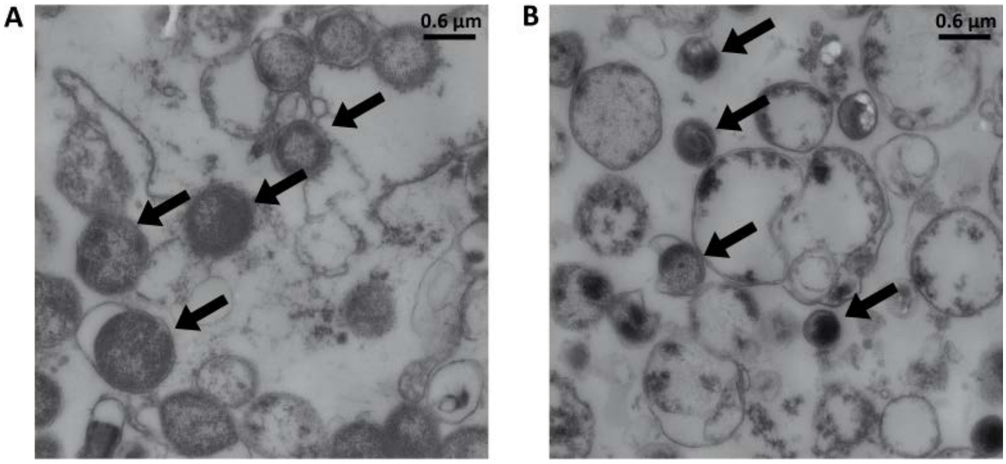

Figure S1: Electron micrographs of purified RB and EB. RB **(A)** and EB **(B)** were purified by differential centrifugation of disrupted infected host cells followed by density gradient centrifugation in a discontinuous sucrose gradient. EB (lower) and RB (upper) fractions were removed from the gradient and washed in SPG buffer. Purity of each fraction was estimated by electron microscopy. Arrows depict RB **(A)** and EB **(B)**, respectively.

Figure S2

A

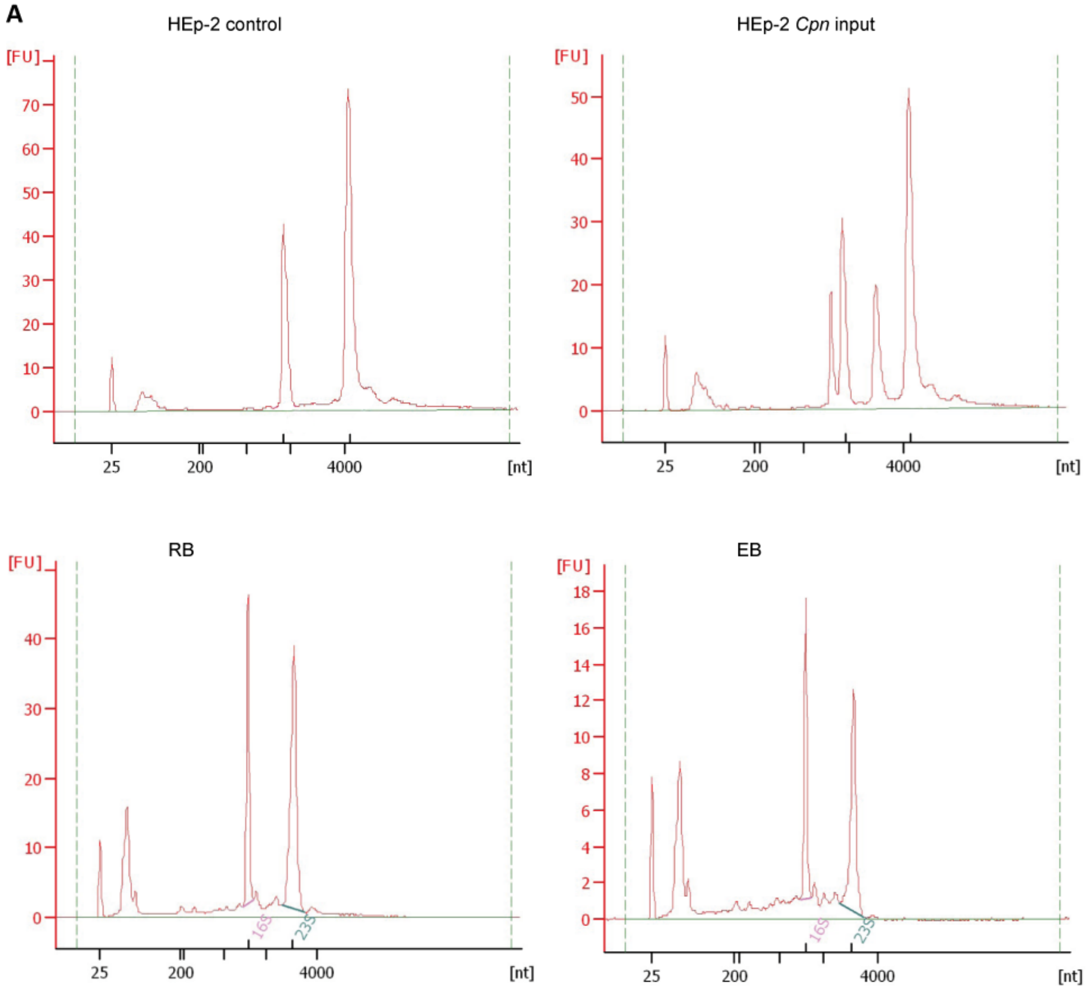

B

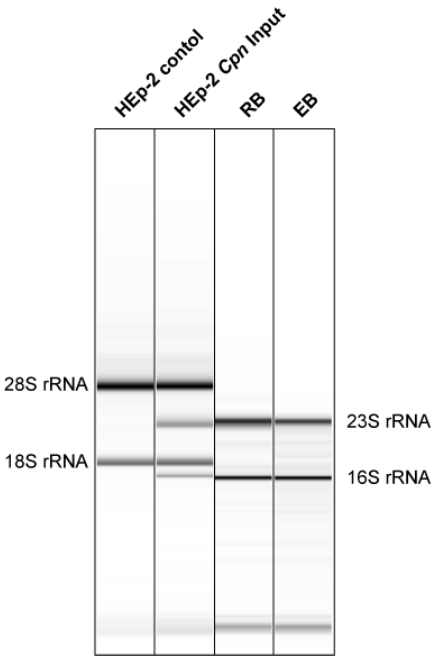

C

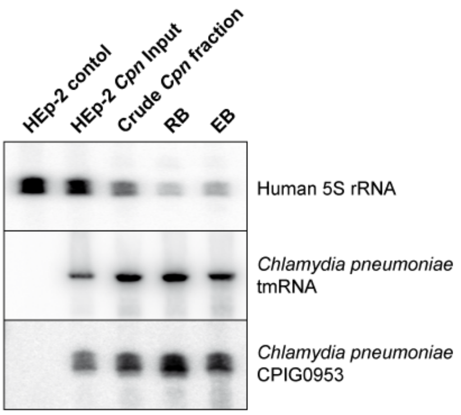

Figure S2: RNA quality determination (previous page). (A) Electropherogram of RNA isolated from uninfected HEp-2 cells, from HEp-2 cells infected with *Cpn*, and from purified RB and EB, respectively, was performed to assess the integrity of the RNA. Eukaryotic ribosomal RNAs are absent in purified RNAs from RB and EB. **(B)** Gel-like image derived from the electropherograms shown in (A). Bacterial (16S and 23S) and human (18S and 28S) ribosomal RNAs are marked. **(C)** Northern Blot analysis of RNA isolated from purified EB and RB was performed with 10 µg of total RNA from uninfected Hep-2 cells (Hep-2 control), HEp-2 cells infected with *Cpn* at MOI of 5, the crude *Cpn* fraction derived from host cell lysis and differential centrifugation (Crude *Cpn* fraction) as well as purified RB and EB derived from gradient centrifugation. Host cell ribosomal 5S RNA is depleted whereas chlamydial RNA is strongly enriched during the purification process.

Figure S3

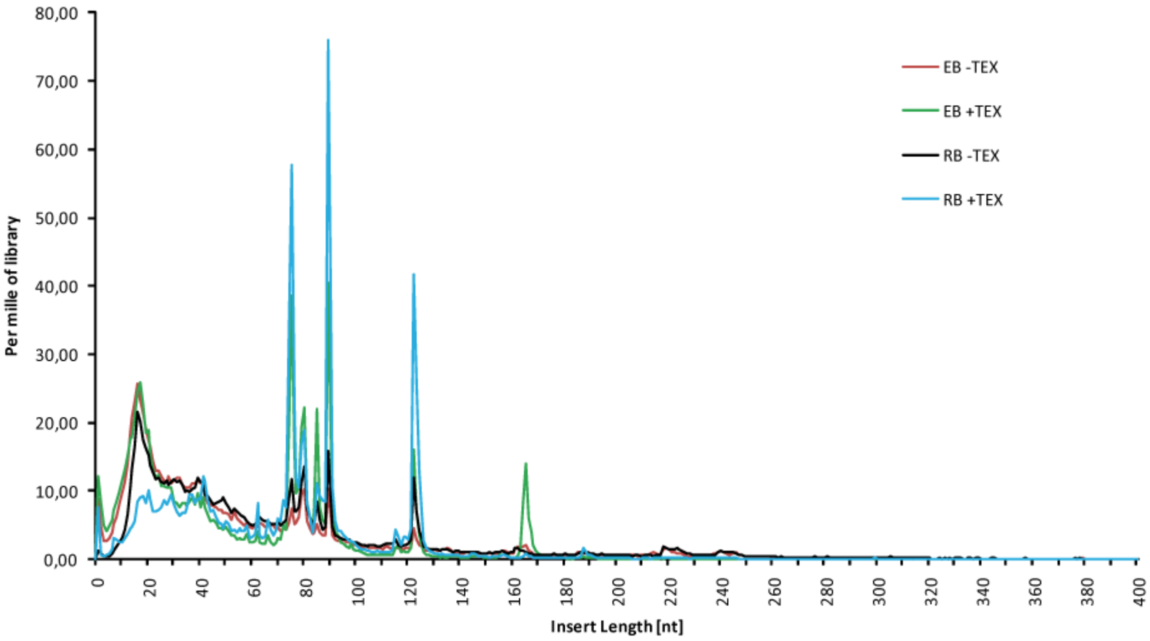

Figure S3: Sequence read length distribution of *Cpn*. Graph showing the length distribution of sequence reads after linker and polyA removal of the four cDNA libraries. Peaks correspond to highly abundant RNA species like tRNAs (70–90 nt).

Figure S4

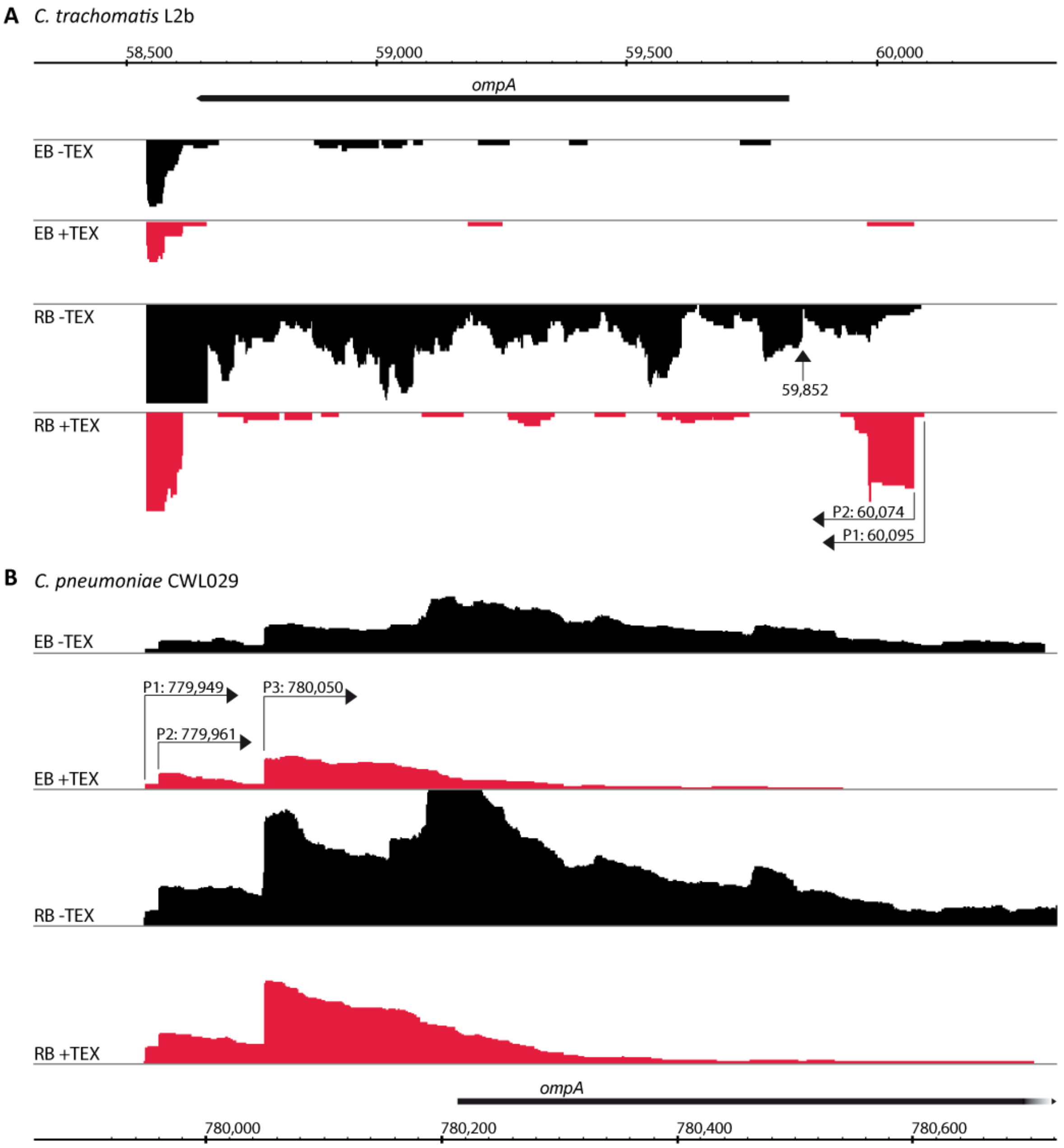

C

```
           -35                                   -10
ompA P1  TATAATGAAA AGAATATACA AAAAAGCTAT AGCTTTCCT
ompA P2  GAATATACAA AAAAGCTATA GCTTTCCTAT AGCTCATAA
ompA P3  GTCTTTACTA TAATAAGAAA AGTTTGATAT GTTTTCGAC
```

D

```
                                                                            P1
Ctr  GAGCGATTTT TTTCGCAAAA ACCAAGAACA TAAAAACATA AAAAGATATA CAAAAATG-G CTCTCTGCTT
Cpn  GAGCCATTTT TAAGG-GGAA CTAAATTATA TATATAATGA AAAGAATATA CAAAAAAGCT ATAGCTTTCC

                 P2
Ctr  TATTGCTAAA TCAGGAGGCG CTTAAGGGCT TCTTCCTGGG ACGAACGTTT ----TTCTTA TCAACTTTAC
Cpn  TATAGCTCAT AACAGAAGTT CTTGGTTGAA ATGTGCGGCT AAAAACACTT AATCTTCTTA TCGTCTTTAC
       P1          P2

Ctr  GAGAATAAGA AAATTTTGTT ATGGTCTCGA GCATTGAACG ACATGTTC -150
Cpn  TATAATAAGA AAAGTTTGAT ATGTTTTCGA CTAATGAGCT GTATGTTC -151
                                        P3
```

Figure S4 (previous page): TSS of the major outer membrane protein gene *ompA*. Sequence read distribution is shown for the *ompA* gene of *Ctr* (minus strand) and *Cpn* (plus strand) for EB and RB and total RNA libraries (black) and libraries enriched for primary TSS (red). Note that the Rho-independent terminator downstream of the *ompA* coding sequence is highly enriched in the sequencing reads due to a thermodynamic highly stable secondary structure **(A)** The *ompA* gene of *Ctr* has a single major TSS at position 60,074 (P2, -248 to translational start), a minor TSS represented by only 1 cDNA read (P1 -269 to TSS), and a processing site at position 59,852 (-25) as earlier described (see text for details). **(B)** Sequencing data suggests three TSS for the *Cpn* ompA gene at the indicated positions P1 to P3 which correspond to positions -266, -254 and -165 relative to the translational start site, respectively, as indicated by arrows. **(C)** Alignment of promoter sequences of three *Cpn* ompA TSS P1 to P3 show poor conservation of -10 and -35 box. **(D)** Alignment of promoter sequences of *Ctr* and *Cpn* with TSS start sites of *Cpn* (on top of alignment) and *Ctr* (below alignment). Interestingly, only P2 is conserved among the two species, even though -10 and -35 promoter regions are partially conserved among P1 and P3.

Figure S5

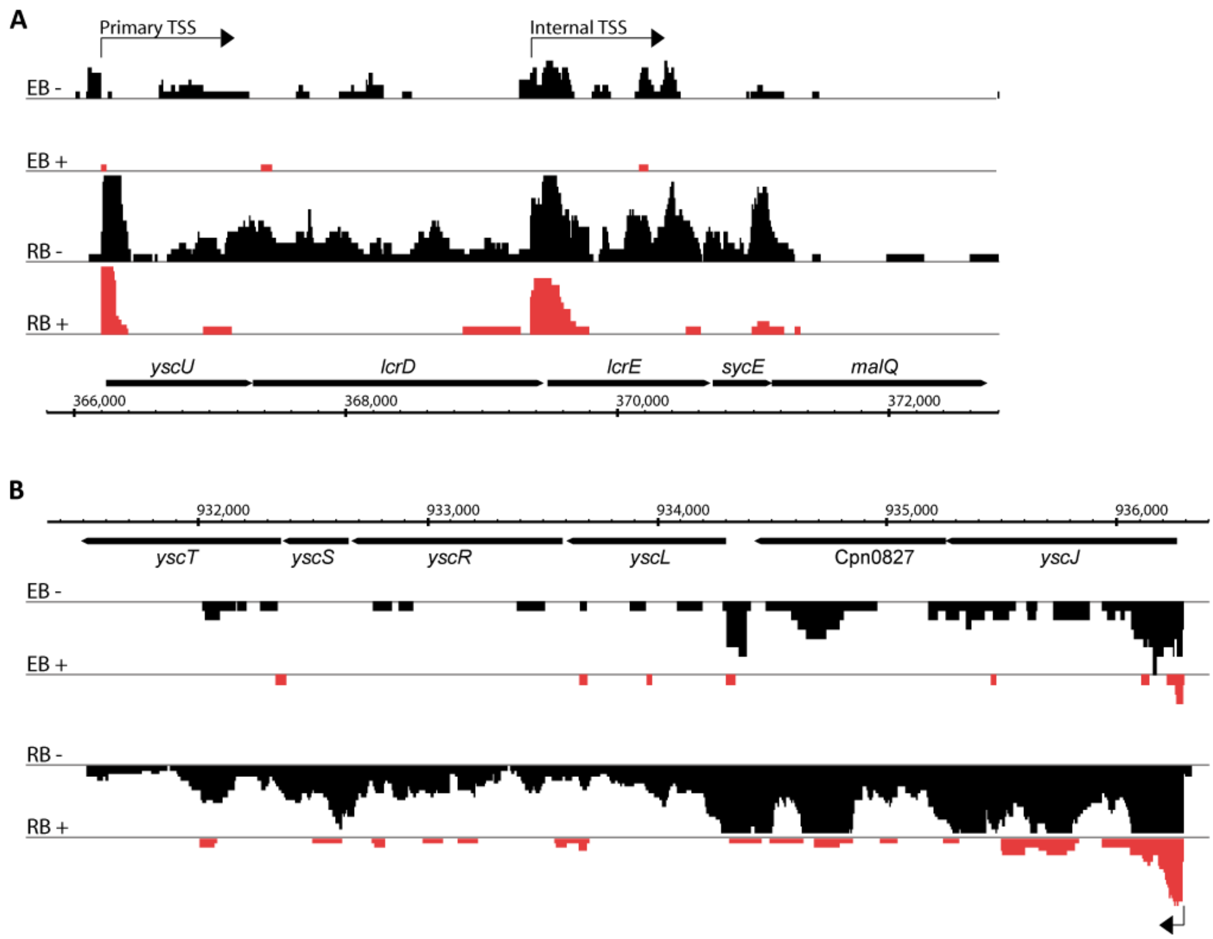

Figure S5: Two operons encoding genes of the type three secretion system in *Cpn*. Polycistronic transcripts can be identified by comparing untreated and TEX-treated cDNA libraries. In the untreated libraries (black bars) sequence reads are distributed among adjacent genes and also cover gene junctions. The TEX-treated libraries (red bars) clearly show the TSS of a polycistronic transcript. **(A)** Sequencing data and Hefty and Stephens (32) suggest that one long transcript contains five genes (*yscU-malQ*). Furthermore, an internal additional TSS belongs to a shorter transcript containing only three genes (*lcrE-malQ*). Black arrows depict TSS. The operon shown in **(B)** contains six genes and is transcribed from a single TSS.

Figure S6

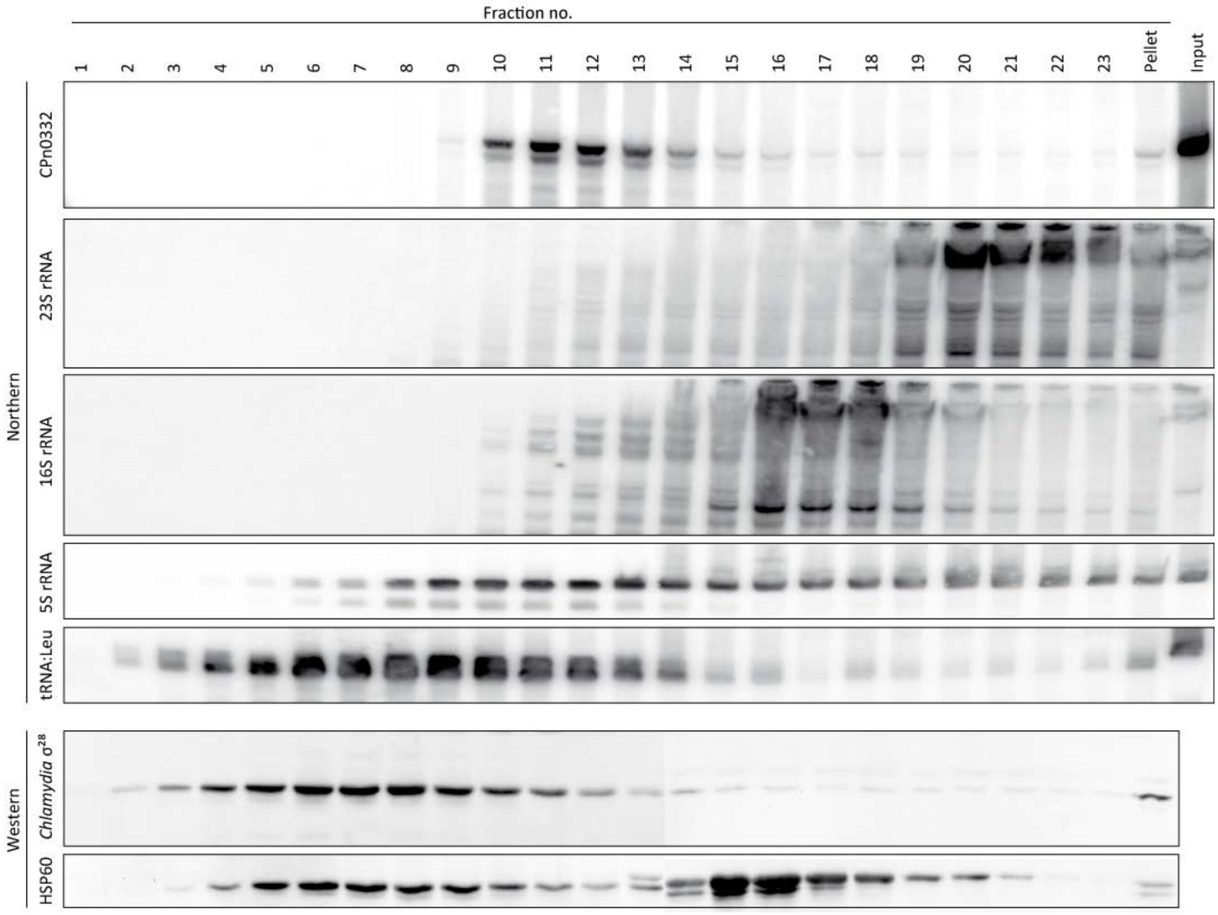

Figure S6: Density gradient fractionation of *Cpn* lysates. *Cpn* whole cell lysate was separated on a 5-40% continuous glycerol gradient. From each of the 24 fractions total RNA was isolated and a Northern blot was performed using probe directed against the abundant RNA encoded by CPn0332, as well as ribosomal RNAs. From the same fractions a Western blot was performed using an antibody directed against chlamydial σ^{28} factor. An association of RNA CPn0332 with σ^{28} RNA polymerase can be excluded since they are located in different fractions of the density gradient. The ubiquitously expressed heat shock protein 60 (encoded by *groEL*) was used a control. Note that, due to the large number of samples, the Western blot picture is assembled from two blots that were treated the same way and in parallel. Fraction number 1 is the lightest on top of the gradient and fraction 24 is the heaviest and contains the insoluble resuspended pellet.

Figure S7

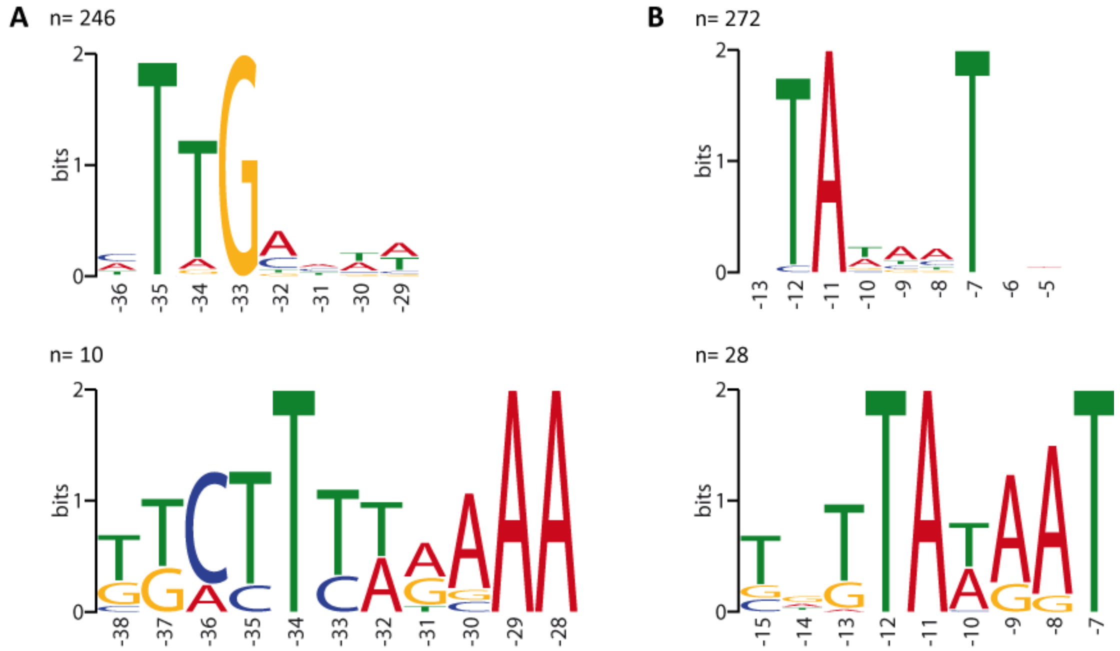

Figure S7: Most prominent sequence motifs of the -35 and -10 boxes of *Cpn*. Promoter sequences were extracted from 531 primary TSS from -28 to -38 **(A)** and from -5 to -15 **(B)** and searched for common sequence motifs using MEME, respectively. The two most prominent motifs are shown in **(A)** for the -35 box and in **(B)** for the -10 box. The number of promoter sequences are given for each motif.

Figure S8

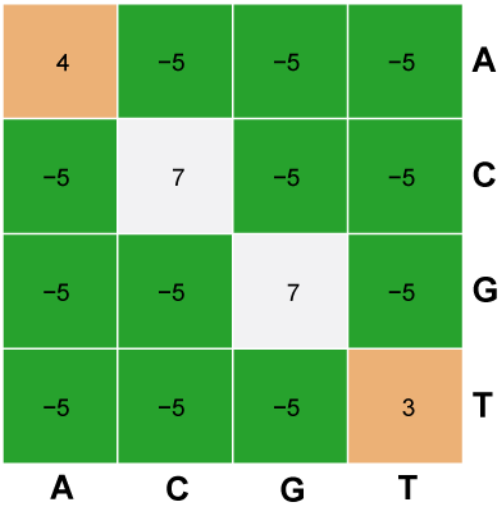

Figure S8: Composition adjusted score matrix. Due to the strong compositional bias in the promoter sequences of *Chlamydia* (AT 70.5% versus GC 29.5%), we derived a composition adjusted score matrix. This matrix is based on the Felsenstein (53) model, where the substitution of any nucleotide by another is proportional to the relative frequency of the substituting nucleotide.

Table S1

Library	Tag FLX library	FLX cDNAs	Tag Titanium library	Titanium cDNAs	Pooled all	cDNAs < 18 nt	cDNAs ≥ 18nt	Total BLAST hits	cDNAs ≥ 18nt but no BLAST hit
**CPEB-**	GTAT	191,720	ACGTGC	204,390	396,110	76,018	320,092	173,808	146,284
**CPEB+**	GTGA	205,526	AGCGTA	200,285	405,811	86,244	319,567	207,239	112,328
**CPRB-**	GTTC	102,238	AGTCAG	216,815	319,053	33,697	285,356	212,974	72,382
**CPRB+**	TCAT	120,587	ATACTG	195,670	316,257	19,528	296,729	260,221	36,508
**Total**		620,071		817,160	1,437,231	215,487	1,221,744	854,242	367,502

Table S1: Numbers of sequence reads of *Cpn* cDNA libraries. Sequence reads were pooled from two sequence runs with FLX and Titanium chemistry, respectively. All reads ≥ 18 nt were blasted against the *Cpn* genome. Sequence reads that gave no BLAST hit are derived from contaminating human host RNA.

Table S2 (Excel spread sheet "Table S2.xls)": Transcription start sites and sequencing read numbers of *Cpn* genes. The exact transcription start sites of the annotated genes including novel non-coding RNAs are listed. For some genes a secondary transcription starts listed which can be the consequence of alternative transcription start sites. Numbers in TSS and location refer to the first nucleotide of the transcript or the annotated gene coordinates, respectively. Genes without TSS are either too low expressed or transcribed as part of a polycistronic transcript. The table gives the following information: First column: indicates the relative location of the TSS to annotated genes (S= sense, AS= antisense, IG= intergenic region, LL= leaderless transcript); TSS: transcription start site; 2nd TSS: secondary TSS located downstream of primary TSS or inside an operon or ORF; Length: length of the corresponding protein in amino acids; Location: start and end coordinates of gene; Strand: sense (+) or antisense (-) strand; Gene: gene name or gene number if no name is available; Synonym: gene number according to NCBI Reference Sequence NC_000922; Product: name of the gene product; EB-, EB+, RB-, RB+: sequence read numbers of the four cDNA libraries derived from either EB or RB total RNA (-) or total RNA enriched for primary transcripts (+). Novel transcripts are coloured in red. Background colour depicts genes that are co-transcribed as operon. Adjacent genes that are transcribed as polycistronic transcript are coloured in green or orange. Genes that are adjacent and co-transcribed but interrupted in the table by genes on the antisense strand are coloured in violet.

Table S3

TSS	Annotation Start	End	Relative location of TSS	Strand	Gene
151,170	151,164	151,781	+6	+	CPn0120 (*gmk*)
297,709	297,152	297,730	-21	-	CPn0264 (*ubiD*)
449,019	449,015	449,713	+4	+	CPn0403 (*yceC*)
461,462	460,218	461,498	-36	-	CPn0415
479,378	477,273	479,462	-84	-	CPn0434
575,352	575,143	575,364	-12	-	CPn0494
582,465	582,457	583,653	+8	+	CPn0501 (*hrcA*)
702,011	701,417	702,025	-14	-	CPn0611 (*coaE*)
856,966	856,957	857,697	+9	+	CPn0760
1,027,604	1,027,595	1,027,825	+9	+	CPn0896

Table S3: Genes that have to be re-annotated. The newly identified TSS have been compared to the translational start sites from the genome annotation. Ten genes have been identified where the TSS is located downstream of the translational start. These genes seem to be wrongly annotated and therefore should be considered for re-annotation.

Table S4

Operon size [genes]	Number of operons	Number of genes
2	129	258
3	61	183
4	30	120
5	11	55
6	6	36
7	1	7
8	3	24
10	2	20
12	2	24
25	1	25
Sum	246	752

Table S4: Operon numbers and lengths of *Cpn*. 246 Operons that encode a total of 752 annotated genes were identified by analysis of deep sequencing data. The operon size listed corresponds to the number of encoded genes. The number of operons corresponds to the number of operons identified in the respective size and the number of genes is the product of operon size and number of operons of that respective size.

Table S5

Position	Start	End	Strand	Putative candidate	Validated Candidate	Probe Name	Probe Sequence	Signal	Theoretic Size [nt]	Northern Size [nt]
AS	28,995	28,435	-	pCpn01		Cpn_sRNA_028.927-	AGGGAGTGACCTTGATCACACAAG	-		
AS	49,662	50,571	+	pCpn02		Cpn_sRNA_049.751+	GGATTCCTCTCACATCCTTGGCT	+	531	400
AS	59,034	58,846	-	pCpn04		Cpn_sRNA_058.998-	TCAAGAGAGCCGGTATCTAGAGGG	-		
AS	78,886	77,712	-	pCpn06		Cpn_sRNA_078.499-	GCTTGGGTGGTTCTCGCTCTACT	+		90,150
AS	94,794	94,609	-	pCpn07		Cpn_sRNA_094.755-	TTATCGTGGAAGTTGGCTCGAAG	-		
AS	102,542	102,308	-	pCpn08		Cpn_sRNA_102.496-	TGTAGTTTGGGGAATCCGACAGA	-		
IG	104,806	104,677	-	pCpn09		Cpn_sRNA_104.773-	TGCAACCCGTTTCTTATTTGTGG	+	130	23
AS	132,469	132,027	-	pCpn10		Cpb_sRNA_132.426-	TGCTTATAGGCCTTCCTTTCATCA	+		200
S	132,303	132,814	+	pCpn11		Cpn_sRNA_132.332+	TAGGGAACGTTAGGGTAGCTGCC	-		
IG	175,970	176,208	+	pCpn12	CPIG0142	Cpn_sRNA_176.193+	GGAACCTCTATAGATCCAACCAGCTC	+	80	80
AS	184,478	183,992	+	pCpn13		Cpn_sRNA_184.426-	TTCACACTTCAGAAGGAAAACAAGG	-		
AS	195,128	195,218	+	pCpn14	CPAS0152	Cpn_sRNA_195.152+	CAAGACAGAAGTATGTCCGAAGCA	+	90	80
AS	237,544	237,186	-	pCpn15		Cpn_sRNA_237.483-	TCGACGAACTTCAGACTTTCGTTT	+	260	220
IG	248,672	248,719	+	pCpn16	CPIG0207	Cpn_sRNA_248.715+	GAAGGCTAAATCACAGGTGGTCC	+	45	~40
AS	251,272	250,976	-	pCpn17		Cpn_sRNA_251.144-	GCAATAACCCCTGCAGGTTTAGC	-		
AS	270,299	270,431	-	pCpn19		Cpn_sRNA_270.341+	TGAGACTCCCGATTTGAAAATCG	-		
AS	278,732	278,895	+	pCpn21		Cpn_sRNA_278.807+	CTGCCGCCTCACAATCTACAATC	-		
AS	282,641	282,703	+	pCpn24		Cpn_sRNA_282.701+	ATTAGGCCTTGCACGAGCTCTCT	+	63	
IG	298,666	298,738	+	pCpn25		Cpn_sRNA_298.729+	CCCCCTAACATAGGGGGTTCTAAA	-		
AS	317,557	317,607	+	pCpn26		Cpn_sRNA_317.601+	AGAAGCTTTCCAGGCTGCCATAC	+	50	50
AS	325,089	325,380	+	pCpn29		Cpn_sRNA_325.142+	CGATGCCTTTTGTAGCTTTGCTTA	-		
AS	330,522	330,471	-	pCpn31		Cpn_sRNA_330.493-	TTTTATTTACAGCAGCCCTGGTCC	-		
AS	333,613	333,433	-	pCpn32	CPAS0294	Cpn_sRNA_333.440-	TGCCCATCCGTAGCTTTATCTTTT	+	110	95
IG	333,613	333,433	-	pCpn32	CPIG0294	Cpn_sRNA_333.553-	GCACGCCGTAATCACTATTGAAAA	+	65	65
AS	333,759	333,823	+	pCpn33		Cpn_sRNA_333.824+	CAGTTAGGAGTGGATCCAAAAGAA	-		
AS	349,722	350,069	+	pCpn35		Cpn_sRNA_349.746+	ATCATTCGACGGTAATCTCATCCA	+	235	120
S	378,551	378,788	-		CPn0332	Cpn_sRNA_378.724-	TAATTACCCAGGCTTCCTGTCGTT	+	240	80-250
AS	389,562	389,909	+	pCpn36		Cpn_sRNA_389.598+	TGGAAGTCTTTTCACGGGTTCTC	-		
AS	410,234	410,104	-	pCpn37		Cpn_sRNA_410.349+	CTCGCGAGTAAATCAAGCCTTTC	-		
IG	410,321	410,359	+	pCpn38		Cpn_sRNA_410.350+	GCTCGCGAGTAAATCAAGCCTTT	-	40	-
AS	428,595	418,822	+	pCpn39		Cpn_sRNA_428.757+	TAGAGTTTCAGGACTTGCGCAGG	-		
IG	445,308	445,452	+	pCpn41	CPIG0397	Cpn_sRNA_445.394+	GGACGCAACCACTAAGAGCTAACA	+	144	140
IG	447,422	447,312	-	SRP_RNA		Cpn_sRNA_447.387-	TCGCAGGTTCCTTCTCTTAAAAGG	+	110	95,110
IG	503,607	503,533	+	pCpn46		Cpn_sRNA_503.534-	AATGGGCAGAGTACGTGATCTCAC	-		
IG	513,197	512,905	-	pCpn47		Cpn_sRNA_513.011+	TTCGTTTGGGAGAATTTCTTGAGA	-		
IG	523,061	522,987	-	pCpn48	CPS0657	Cpn_sRNA_523.009-	GATTGTGAAAGAGGCTTTTGCCC	+	73	70
IG	548,000	548,167	+	pCpn50		Cpn_sRNA_548.042-	CGCAAGGGAAACTTGAAGAGTTTT	-		
AS	578,934	579,257	+	pCpn51		Cpn_sRNA_578.979+	TTCTGTTGGGTGTTGTCCTTGGT	-		
AS	589,097	588,471	-	pCpn52		Cpn_sRNA_589.023-	ATCGGCATCGATTATGCTCAAGA	-		
IG	654,455	654,421	-	pCpn53	CPIG0564	Cpn_sRNA_654.431-	TCCACCTTCAAAGAGAGCTCTTTA	+	35	35,40
AS	660,667	660,351	-	pCpn54		Cpn_sRNA_660.507-	GTCATTAGAGGGACCTCCGGAGA	-		
AS	671,049	671,467	+	pCpn55		Cpn_sRNA_671.075+	AGATGCCACGCACACACTACGTA	-		
IG	692,664	692,320	-		CPn0600.1	Cpn_sRNA_692.520-	GCAACAGGAAGCACAAGAGCTAAA	+	344	420
S	738,367	738,277	-	pCpn57	CPS0657	Cpn_sRNA_738.296-	CTTCCGCGATAGTATCTCCGAGAT	+	88	90
IG	767,056	767,090	+	pCpn58		Cpn_sRNA_767.081+	TTCCATGGATATGGAATGGCTTTT	+	34	130
IG	775,135	775,010	-	pCpn59	CPIG0692	Cpn_sRNA_775.105-	CAGCATGGAATACACTAAGGCGC	+	122	120
IG	784,727	784,788	+	pCpn60		Cpn_sRNA_784.787+	GAGCAATCTTTTCCCTCTCTATGA	+	61	110
IG	786,909	786,802	-	pCpn61	CPIG0701	Cpn_sRNA_786.828-	TACGGCCGACTCTACATCTTCTTG	+	106	100
S	818,360	818,173	-	pCpn62		Cpn_sRNA_818.395-	GCCTCCAATCCTTGAGCAGAAAG	-		
IG	844,006	844,038	+	pCpn64		Cpn_sRNA_844.029+	TGAAGATAAAATCTTCATCCCCGA	+	31	30
IG	858,486	858,441	-	pCpn66		Cpn_sRNA_858.452-	CCCTCCTAAAGTATTAGAAGGGGG	+	45	450
IG	1,088,328	1,088,465	+	pCpn68	CPIG0953	Cpn_sRNA_1.088.374+	TAAAGGGTTGTGAGCTCGTGAAAA	+	137	130
IG	1,088,976	1,088,925	-	pCpn69	CPIG0954	Cpn_sRNA_1.088.955-	CTTTCGAAAGCCATGTGCTTATGT	+	50	52
AS	1,136,800	1,136,420	-	pCpn72		Cpn_sRNA_1.136.753-	TTTATGGAAAGAACGCCGTCCTT	-		
AS	1,206,657	1,206,332	-	pCpn74		Cpn_sRNA_1.206.564-	GCGATGACTTTCCAAGTTAGCGA	+	320	65,150,240

Table S5: Northern blot results of putative sRNAs of *Cpn*. 54 out of 75 sRNA candidates have been tested for expression by Northern blotting. 13 of these could be positively verified since the detected band corresponded to the theoretical size of the sRNA candidate calculated from the sequencing data. These verified sRNAs were named according to the protein coding gene encoded upstream, antisense or sense, respectively. A highly abundant sRNA was found to be encoded in ORF CPn0332. A sRNA candidate that turned out to encode a protein coding gene missing in the annotation was named CPn0600.1. The signal recognition RNA (SRP RNA) was used as positive control. Columns are labelled as follows:

Position: AS = antisense to annotated ORF, IG = intergenic region, S = sense to annotated ORF; Start/End, genomic coordinates of sRNA; Strand: sense (+) or antisense (-) strand encoding sRNA; Putative Candidate: preliminary name; Validated Candidate: final name of validated sRNA; Probe Name: name of oligonucleotide used for Northern hybridization, contains coordinate of first nucleotide and strand; Probe Sequence: nucleotide sequence of the probe in 5' to 3' direction; Signal: indicates whether a signal was detected in Northern hybridization; Theoretic Size: length in nt calculated from sequencing data; Northern Size: length in nt detected by Northern hybridization.

Table S6 (Excel spread sheet "Table S6"): List of relative gene expression in EB and RB. All genes with at least 20 sequence reads in total and a maximal read count lower than 1,000 were considered. Out of 1,012 genes in the analysis 288 were classified as differentially expressed (multiple testing corrected p-values <0.05, twofold change, minimum 20 sequence reads in total), whereof 91 (31,6%) are more abundant in EB and 197 (68.4%) are up-regulated in RB. Fold changes have been derived from the common odds ratios from the Mantel-Haenszel test.

Supplemental Methods:

Preparation of *C. pneumoniae* Lysates

Four 75 cm² cell culture flasks with Hep-2 cells at a confluency of approximately 80% were infected with *C. pneumoniae* at a MOI of 5 for 40h hours. Cells were washed with ice cold PBS and collected by scraping on ice. Cells were pooled in a total volume of 20 ml cold PBS and disrupted by adding 5 ml of glass beads (1 mm diameter) and vortexing for 5 min at maximum speed with. Cell debris was removed by pelleting for 10 minutes at 1,000 rcf and 4°C. *Chlamydia* containing supernatant was centrifuged at 25,000 rcf for 20 minutes at 4°C to pellet bacteria and washed once with lysis buffer. Bacteria were resuspended in 2 ml lysis buffer containing Triton X-100 at a final concentration of 0.2% and portioned into two Lysis Matrix B tubes. Bacteria were disrupted by homogenization for 5x 20 seconds at 6.5 m/s with dry ice cooling in a FastPrep homogenizer (MP Biomedicals). The lysates were cleared by centrifugation at 14,000 rcf at 4°C for 10 minutes.

Fractionation of Lysates on a Density Gradient

Molecular biology grade glycerol was used to prepare 1% and 40% stock solutions in lysis buffer containing 0.2% Triton X-100. The solutions were filtered through a 0.2 µm pore size filter. Continuous 1-40% glycerol gradients were formed in 12 ml ultracentrifuge tubes using a gradient maker device and a peristaltic pump. The cleared lysate was layered on top of the gradient and separated by centrifugation for 17h at 100,000 rcf and 4°C.The gradient was fractionated into 24 fractions of 500 µl each whereby the last fraction contained the resuspended pellet. 200 µl of each fraction were used for protein detection by Western blotting. The remaining 300 µl lysate from each fraction were transferred to PhaseLock tubes and RNA was isolated by the phenol/chloroform method. One volume (300 µl) of P:C:I was added to the fractions followed by vigorous shaking for 30 seconds. Phases were separated by centrifugation at 14,000 rcf for 15 minutes at 4°C. The aqueous phases were transferred to fresh 1.5 ml tubes and 1.5 µl of GlycoBlue were added to each fraction as carrier to improve precipitation. RNA was precipitated by addition of 2.5 volumes ethanol containing 0.1 M sodium acetate, shaking, and incubation over night at -20°C. Subsequently RNA was pelleted by centrifugation for 20 minutes at 20,000 rcf and 4°C. The pellets were washed with 75% ethanol and air dried for 5 to 10 minutes. RNA was dissolved in 30 µl water and concentrations were estimated spectrophotometrically by a Nanodrop device (Thermo).

SDS-PAGE and Western Blotting

Proteins were separated on the basis of mass by electrophoresis in a polyacrylamide gel under denaturing conditions with sodium dodecyl sulphate (SDS-PAGE). Proteins in fractions from the glycerol gradient were denatured in 4x SDS-loading buffer by heating at 95°C for 7 min. The denatured proteins were electrophoresed in a 12% polyacrylamide gel. Proteins were blotted onto PVDF membranes using a semidry electro blotter. For immunodetection, the membrane was blocked in TBST buffer containing 5% milk and 1% BSA for 1 h at RT. The primary antibody was diluted in TBST buffer containing 1% BSA and added to the membrane. The membrane was incubated with the primary antibody overnight at 4°C on a shaker. After incubation, the membrane was washed again three times for 5 min with TBST

buffer. The appropriate peroxidase-conjugated secondary antibody was diluted in TBST buffer and added to the membrane. The membrane was incubated with the secondary antibody at RT for 1 h and washed three times with TBST buffer. This step was followed by the standard enhanced chemiluminescence reaction (ECL system).
